# Defining the features and duration of antibody responses to SARS-CoV-2 infection associated with disease severity and outcome

**DOI:** 10.1126/sciimmunol.abe0240

**Published:** 2020-12-07

**Authors:** Katharina Röltgen, Abigail E. Powell, Oliver F. Wirz, Bryan A. Stevens, Catherine A. Hogan, Javaria Najeeb, Molly Hunter, Hannah Wang, Malaya K. Sahoo, ChunHong Huang, Fumiko Yamamoto, Monali Manohar, Justin Manalac, Ana R. Otrelo-Cardoso, Tho D. Pham, Arjun Rustagi, Angela J. Rogers, Nigam H. Shah, Catherine A. Blish, Jennifer R. Cochran, Theodore S. Jardetzky, James L. Zehnder, Taia T. Wang, Balasubramanian Narasimhan, Saurabh Gombar, Robert Tibshirani, Kari C. Nadeau, Peter S. Kim, Benjamin A. Pinsky, Scott D. Boyd

**Affiliations:** 1 Department of Pathology, Stanford University School of Medicine, Stanford, CA, USA.; 2 Stanford ChEM-H and Department of Biochemistry, Stanford University School of Medicine, Stanford, CA, USA.; 3 Department of Structural Biology, Stanford University, Stanford, USA.; 4 ATUM, Newark, CA, USA; 5 Department of Medicine, Division of Pulmonary, Allergy and Critical Care Medicine, Stanford University, Stanford, CA, USA.; 6 Sean N. Parker Center for Allergy and Asthma Research, Stanford, CA, USA.; 7 Stanford Blood Center, Palo Alto, CA, USA.; 8 Department of Medicine, Division of Infectious Diseases and Geographic Medicine, Stanford University, Stanford, CA, USA.; 9 Stanford Center for Biomedical Informatics Research, Stanford University, Stanford, California, USA; 10 Chan Zuckerberg Biohub, San Francisco, CA, USA.; 11 Department of Bioengineering, Stanford University, Stanford, CA, USA.; 12 Department of Microbiology and Immunology, Stanford University, Stanford, CA, USA.; 13 Department of Statistics, Stanford University, Stanford, CA, USA.; 14 Department of Biomedical Data Sciences, Stanford University, Stanford, CA, USA.

## Abstract

SARS-CoV-2-specific antibodies, particularly those preventing viral spike receptor binding domain (RBD) interaction with host angiotensin-converting enzyme 2 (ACE2) receptor, can neutralize the virus. It is, however, unknown which features of the serological response may affect clinical outcomes of COVID-19 patients. We analyzed 983 longitudinal plasma samples from 79 hospitalized COVID-19 patients and 175 SARS-CoV-2-infected outpatients and asymptomatic individuals. Within this cohort, 25 patients died of their illness. Higher ratios of IgG antibodies targeting S1 or RBD domains of spike compared to nucleocapsid antigen were seen in outpatients who had mild illness versus severely ill patients. Plasma antibody increases correlated with decreases in viral RNAemia, but antibody responses in acute illness were insufficient to predict inpatient outcomes. Pseudovirus neutralization assays and a scalable ELISA measuring antibodies blocking RBD-ACE2 interaction were well correlated with patient IgG titers to RBD. Outpatient and asymptomatic individuals’ SARS-CoV-2 antibodies, including IgG, progressively decreased during observation up to five months post-infection.

## Teaser

Illness severity in COVID-19 correlates with specificity of serological responses, but antibody levels decrease in most patients.

## INTRODUCTION

A novel coronavirus first described in Wuhan, China in December 2019 (*1*) has led to a coronavirus disease (COVID-19) pandemic and a global economic shutdown amid unprecedented social distancing measures. The clinical spectrum of COVID-19 ranges from asymptomatic infection and mild upper respiratory tract illness in the majority of patients, to severe viral pneumonia with respiratory failure, multiorgan failure, and death (*2*–*4*). Older adults and people with serious underlying health conditions are at greatest risk for severe illness and death (*5*–*8*). Host immune responses may be one of the most important determinants for disease progression and outcome, but this remains to be established.

The virus causing COVID-19 belongs to the *Sarbecovirus* subgenus (genus *Betacoronavirus*) together with the severe acute respiratory syndrome coronavirus (SARS-CoV) and has been designated SARS-CoV-2 (*9*). Coronaviruses contain four structural proteins, including spike, envelope, membrane, and nucleocapsid (N) proteins. The spike surface glycoprotein contains the receptor binding domain (RBD), which binds strongly to human ACE2 receptors (*1*, *10*), and plays a major role in viral attachment, fusion of viral and host membranes, and entry of the virus into host cells (*11*). Most individuals infected with SARS-CoV-2 develop antibodies to the spike and N proteins, which are therefore used as antigens in clinical serology assays. The spike protein is an important target for neutralizing antibodies, as they can prevent viral entry into host cells (*12*, *13*). Current information on the role of antibodies in viral clearance and modulation of disease severity as well as the durability of these responses following primary infection is limited or controversial. Improved understanding of humoral immunity to SARS-CoV-2 is needed to inform strategies for vaccination and the use of therapeutics in the form of neutralizing antibodies or convalescent plasma. Reports about the longevity of antibody titers to SARS-CoV-2 are not in full agreement, with some finding a rapid waning of virus-specific IgG antibodies by approximately 3 months after infection (*14*, *15*), and others emphasizing stable titers detected over several weeks or several months (*16*–*18*). Virus-specific antibody responses appear to be elevated in COVID-19 patients with severe disease as opposed to asymptomatic or mildly ill individuals, raising concerns about the effectiveness of antibody responses to SARS-CoV-2. A suggestion that the quality rather than quantity of antibodies may predict the outcome of infection is provided by a recent report applying a panel of serological assays to COVID-19 patients who convalesced or died (*19*). Nonhuman primates challenged with SARS-CoV-2 after vaccination with spike-based DNA vaccines developed neutralizing antibodies and immune correlates of protection, suggesting that antibody responses may be more effective in preventing than resolving disease (*20*).

We performed a comprehensive analysis of SARS-CoV-2 RBD, S1 and N protein specific antibodies, and RBD-ACE2 blocking antibodies, spike-pseudotyped virus neutralization, and viral RNA measurements in nasopharyngeal swab and plasma samples of individuals infected with SARS-CoV-2. A total of 254 individuals (79 inpatients and 175 outpatients or asymptomatic individuals) were studied. RBD-ACE2 blocking antibodies and spike-pseudotyped lentivirus neutralization were well-correlated with IgG specific for RBD. Notably, higher ratios of IgG antibodies targeting S1 or RBD compared to N were strongly associated with clinically milder infection. Viral RNAemia decreased to undetectable levels rapidly once plasma antibodies and RBD-ACE2 blocking activity appeared. Outpatients and asymptomatic individuals show substantial and progressive decreases in SARS-CoV-2 specific antibodies after the first month of documented infection.

## RESULTS

### Study design and patient demographics

A total of 254 individuals with a positive SARS-CoV-2 real-time RT-PCR (rRT-PCR) nasopharyngeal swab test were included in the study (fig. S1). Study subjects were identified either after routine serology testing or occupational health screening in the Stanford Health Care Clinical Laboratories for anti-SARS-CoV-2 RBD IgM and IgG antibodies (136 asymptomatic individuals or outpatients), or after they reported to Stanford Health Care-associated clinical sites with symptoms of COVID-19. This included 24 outpatients, 35 hospitalized patients who were not admitted to the intensive care unit (ICU), and 20 ICU inpatients who survived their illness. To evaluate serological responses associated with patient mortality, we also analyzed specimens from 25 patients who died of COVID-19 (one outpatient, seven admitted non-ICU, and seventeen admitted ICU patients). Of patients who were treated in the ICU, 26 of 37 (70%) required mechanical ventilation, including 15 patients who died.

**Fig. 1:**
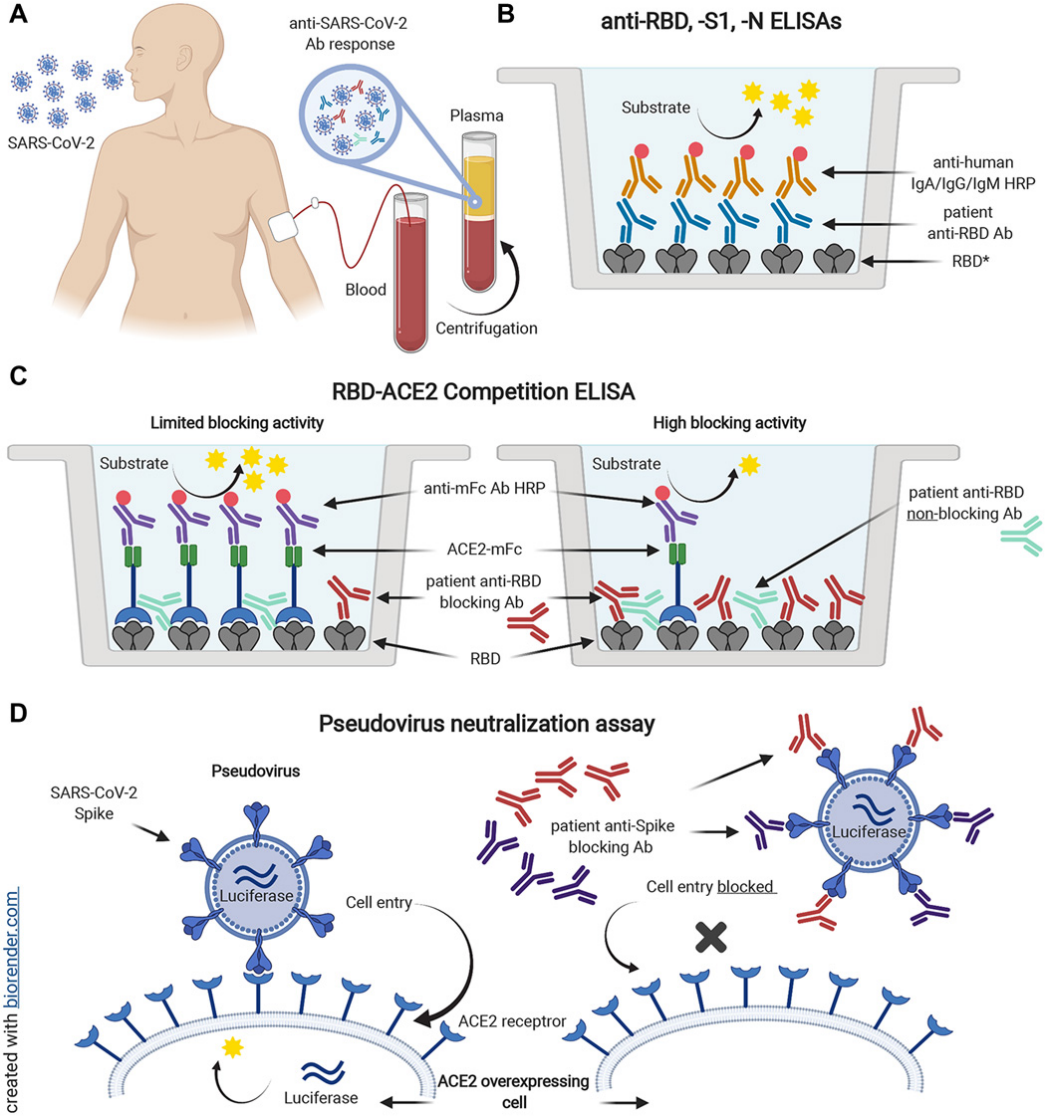
Serological testing of plasma from SARS-CoV-2 rRT-PCR+ individuals. Plasma samples from SARS-CoV-2 rRT-PCR-positive individuals (**A**) were analyzed for the presence of antibodies binding to SARS-CoV-2 spike RBD (**B**). *Plasma was also tested for antibodies specific for SARS-CoV-2 S1 and N proteins, and SARS-CoV RBD. In addition, samples were tested for antibodies blocking the interaction of ACE2 and RBD in an ACE2 competition ELISA (**C**). Spike SARS-CoV-2 pseudotyped lentiviral neutralization assays were performed on selected plasma samples (**D**). Spike-mediated virus entry into HeLa cells overexpressing the ACE2 receptor was measured via luciferase reporter activity.

Demographic and clinical characteristics of patients stratified by disease status are presented in Table 1. The percentage of males, and those with comorbidities of hypertension or diabetes mellitus increased with patient disease severity. Outpatients and asymptomatic individuals were younger and had the lowest levels of obesity compared to more severely ill patients. Notably, levels of viral RNA measured by rRT-PCR of nasopharyngeal swabs at diagnosis showed a progressive increase (lower rRT-PCR cycle threshold, Ct) with disease severity; patients who died had the highest viral loads (fig. S2). Plasma samples for in-depth serological testing were available from all inpatients and 86 of the 160 outpatient survivors. Demographic and clinical characteristics of outpatients and asymptomatic individuals with and without plasma availability are presented in table S1. A total of 828 samples were analyzed with ELISAs measuring IgM, IgG and IgA specific for SARS-CoV-2 RBD, S1 or N, as well as the RBD-ACE2 blocking assay (Fig. 1A-C). Representative samples were also tested for antibody cross-reactivity to SARS-CoV RBD, viral RNA in the blood (RNAemia), and neutralization of spike-pseudotyped lentivirus (Fig. 1D). We also tested 45 plasma specimens from a validation cohort of 14 asymptomatic and outpatient SARS-CoV-2 rRT-PCR-positive individuals with monthly prospective sample collection for up to four months post-enrollment.

**Table 1:** Patient Demographic and Clinical Characteristics.

**Characteristics**		**Outpatients and asymptomatic individuals (n=160)**	**Admitted, non-ICU** **(n=35)**	**Admitted,** **ICU** **(n=20)**	Deceased* (n=25)
Age, median (IQR)		41 (32-56)	55 (40-71)	44 (37-63)	76 (64-85)
Sex (%)	Female	101 (63)	20 (57)	10 (50)	10 (40)
	Male	59 (37)	15 (43)	10 (50)	15 (60)
Diagnostic rRT-PCR Ct, median (IQR)		29.7 (21.8-37.3)	30.6 (21.1-35.4)	25.2 (22.5-29.9)	20.4 (16.5-28.8)
Comorbidities, number of individuals (% present)	Obesity	43 (27 NA) (32)	15 (1 NA) (43)	11 (55)	10 (40)
	Hypertension	30 (19)	8 (23)	7 (35)	19 (76)
	DM	16 (10)	7 (20)	7 (35)	14 (56)
Symptoms on presentation, N of individuals (% present)	Cough	93 (58)	27 (77)	17 (85)	15 (60)
	Fever	72 (45)	23 (66)	15 (75)	11 (44)
	SOB	30 (19)	25 (71)	15 (75)	18 (72)
	Myalgia	60 (38)	15 (43)	11 (55)	3 (12)
	GI	29 (18)	19 (54)	10 (50)	5 (20)
	Fatigue	56 (35)	17 (49)	6 (30)	7 (28)
	Chills	29 (18)	10 (29)	8 (40)	3 (12)
	Headache	37 (23)	6 (17)	5 (25)	1 (4)
Mechanical ventilation (%)		0	0	11 (55)	15 (60)
Length of hospital stay, median days (IQR)		NA	5 (2-8)	17 (9-49)	15 (5-29)
Number of plasma specimens per patient, median (IQR)		1 (1-2)	5 (4-8)	13 (8-21)	8 (3-11)

ICU, intensive care unit; IQR, inter quartile range; rRT-PCR Ct, real-time reverse transcription polymerase chain reaction cycle threshold; NA, not available; DM, diabetes mellitus; SOB, shortness of breath; GI, gastrointestinal. *Of the 25 deceased patients, one, seven, and seventeen were categorized as outpatient, admitted non-ICU, and admitted ICU, respectively. The fourteen individuals that were part of the validation cohort were not included in this table.

### Anti-RBD, S1 and N antibody responses and duration are associated with disease severity

**Fig. 2:**
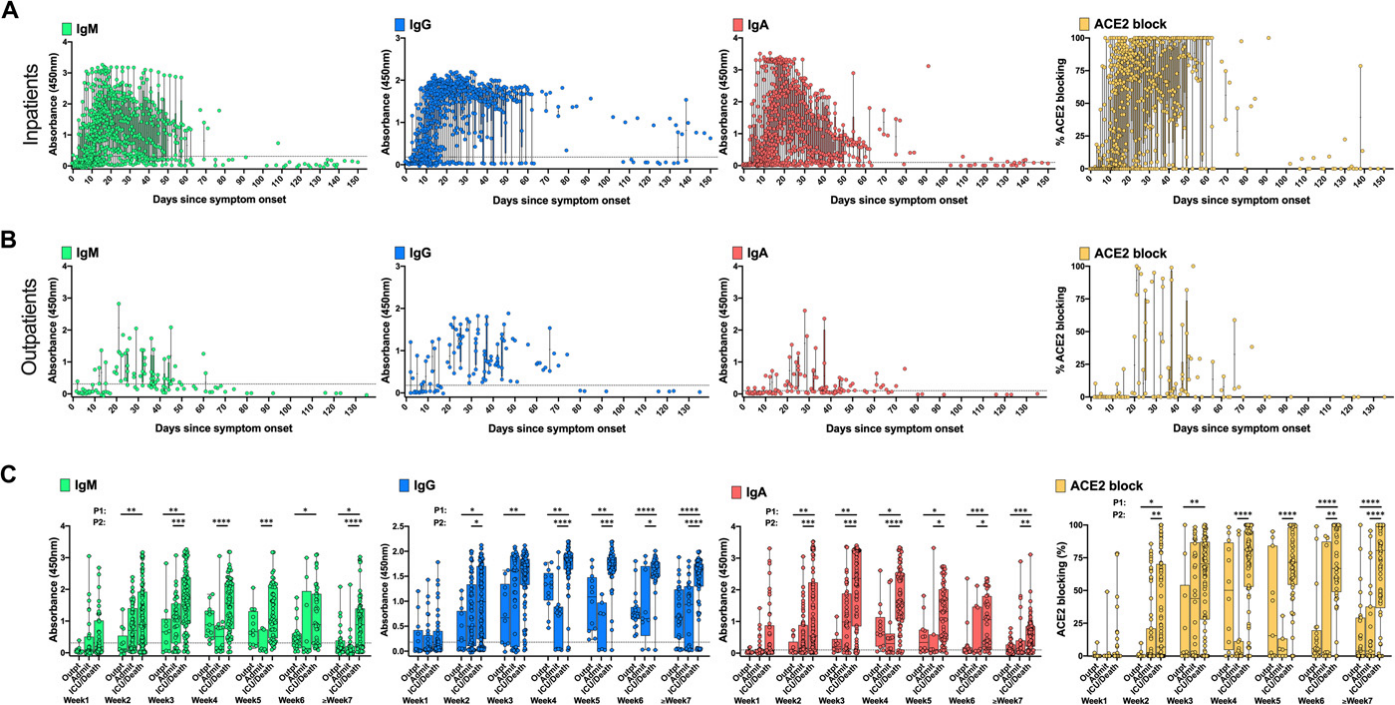
Development of anti-SARS-CoV-2 RBD antibody responses in COVID-19 patients. 828 longitudinal plasma samples collected from 80 COVID-19 inpatients and deceased individuals (714 samples) (**A**) and 86 outpatients (114 samples) (**B**) were tested by ELISA at a dilution of 1:100 for the presence of SARS-CoV-2 RBD-specific IgM, IgG, and IgA antibodies and for antibodies blocking binding of ACE2 to RBD. ELISA data stratified by the 86 outpatients (Outpt), 35 hospitalized patients who did not require ICU care (Admit), and the 20 ICU patients and 25 patients who died, from week 1 to ≥ 7 weeks post-onset of symptoms (**C**). Boxes indicate the interquartile range and whiskers show the minimum and maximum values for each group. Dotted lines denote the assay cutoff. Mean values for duplicate measurements are shown. Statistical testing comparisons are P1 = Outpt vs Admit/ICU/Deceased, P2 = Admit vs ICU/Deceased, by two-sided Wilcoxon rank sum test. * P < 0.05, ** P < 0.01, *** P < 0.001, **** P < 0.0001. Data for 14 samples from 2 patients (one admitted non-ICU and one deceased patient) are not plotted because the time of symptom onset was unknown. Mean ELISA OD_450_ values of duplicate measurements are shown for each sample.

Lower antibody responses in patients with mild symptoms compared to those with more severe disease have been reported for other coronavirus infections, such as MERS-CoV (*21*–*23*) as well as for SARS-CoV-2 (*15*, *24*). The detection rate of RBD-binding antibodies at one-week intervals after symptom onset are shown in table S2; most individuals seroconverted by week 2 post-onset of symptoms (Fig. 2A, B). Positivity rates for RBD IgM, IgG and IgA reached their maximum at weeks 4, 6 and 5, respectively, with most patients negative for IgM and IgA after 12 weeks, while RBD IgG levels showed a slower, but progressive decline, or a continued negative result in those who failed to generate IgG at earlier time points (Fig. 2A, fig. S3, and table S2). Outpatients had lower titers of RBD IgM, IgG, and IgA compared to inpatients, and a more rapid decline of titers (Fig. 2B, fig. S4). Patients who required ICU care, and those who died, developed and maintained the highest levels of IgM, IgG and IgA, as well as RBD-ACE2 blocking antibodies, throughout the time course (Fig. 2C).

S1-specific IgM, IgG, and IgA showed antibody kinetics that were very similar to those seen for RBD (figs. S3 to S5 and table S2). While N-specific IgG responses showed high positivity rates with antibody kinetics similar to those for RBD and S1, IgM antibody responses to N were strikingly low in most patients (figs. S4 and S6 and table S2). There was no consistent difference in RBD, S1, and N antibody titers or RBD-ACE2 blocking antibodies between ICU patients who survived compared to patients who died (fig. S7).

We further evaluated the breadth of antibody responses in different disease severity categories by testing for SARS-CoV RBD binding. Most monoclonal antibodies targeting SARS-CoV RBD fail to bind SARS-CoV-2 RBD, indicating distinct antigenicity despite sequence and structural similarity of the two proteins (*25*, *26*). Nine of 13 ICU patients, three of 25 admitted non-ICU patients (fig. S8A) and five of 82 outpatients (fig. S8B), developed SARS-CoV RBD IgG titers during the course of their infection. The time course of anti-SARS-CoV RBD positivity in serial samples from individual patients did not always mirror anti-SARS-CoV-2 RBD IgG responses, suggesting limited clonal or oligoclonal B cell responses with this broader reactivity within the overall polyclonal anti-SARS-CoV-2 serological response (figs. S8C, D).

### Neutralizing antibodies are increased in inpatients compared to outpatients, and correlate with RBD-ACE2 blocking and RBD IgG titers

**Fig. 3:**
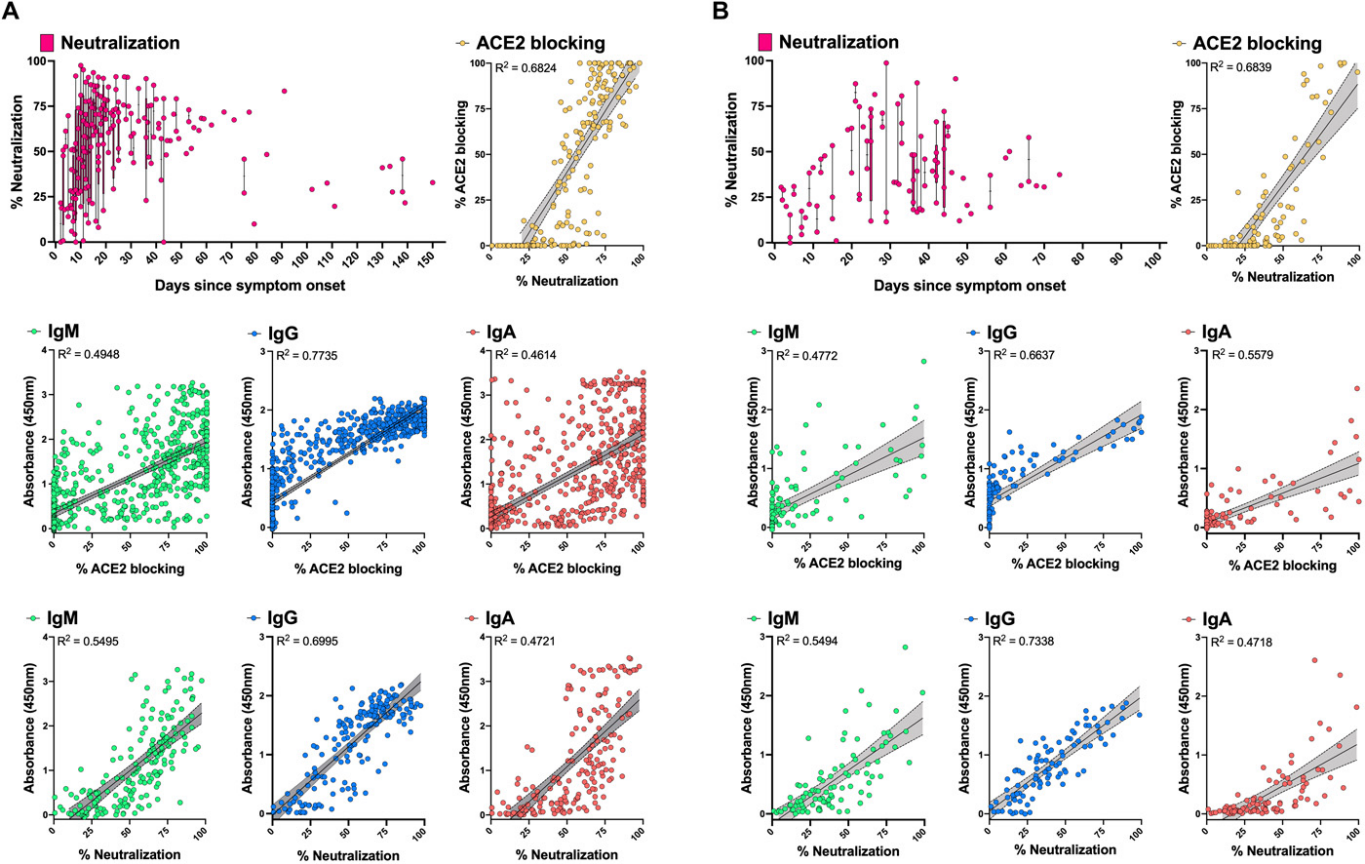
Correlation of spike-pseudotyped viral neutralization, RBD-ACE2 blocking and RBD-specific serology results. Plasma samples from inpatients (n = 188) (**A**) and outpatients (n = 96) (**B**) collected at different time points post-onset of symptoms were tested at a dilution of 1:1250 for their pseudovirus neutralization activity. Correlations with RBD IgM, IgG, IgA (1:100 diluted plasma samples), and RBD-ACE2 blocking ELISA (1:10 diluted plasma samples) data were assessed with simple linear regression and 95% confidence bands (grey shading) of the best-fit line. Correlations between RBD IgM, IgG, and IgA data and RBD-ACE2 blocking ELISA results were done on the full sample set from inpatients (n = 714) and outpatients (n = 114). Plots show mean ELISA OD_450_ values of duplicate measurements and average percent neutralization from duplicate testing in each of two replicate experiments.

Antibody neutralization of live SARS-CoV-2, or of pseudotyped viruses such as lentiviruses expressing the SARS-CoV-2 spike may represent the most physiologically relevant surrogates for humoral immunity in vivo, but are poorly scalable due to restrictive biosafety requirements for SARS-CoV-2 and less easily standardized assay components and protocols, compared to tests using purified proteins. We tested SARS-CoV-2 spike-pseudotyped lentivirus neutralization (*27*) in HeLa cells overexpressing ACE2 (*28*) by inpatient and outpatient samples, and evaluated correlations with the more scalable ELISAs for RBD, S1 and RBD-ACE2 blocking (Fig. 3A, B). As with RBD-ACE2 blocking, neutralizing antibody activity was higher in inpatients compared to outpatients, and began to decrease after about one month after symptom onset. Neutralization was well-correlated with RBD IgG titers and RBD-ACE2 blocking (linear regression coefficient of determination R^2^ of 0.6995 and 0.6824 for inpatients; 0.7338 and 0.6839 for outpatients). The RBD-ACE2 blocking assay was less sensitive than neutralization or RBD IgG ELISA. RBD IgM and IgA ELISA results were much more variable and did not correlate as well with neutralization or RBD-ACE2 blocking compared to RBD IgG.

### Validation of decreasing antibody responses in mildly ill and asymptomatic individuals

**Fig. 4:**
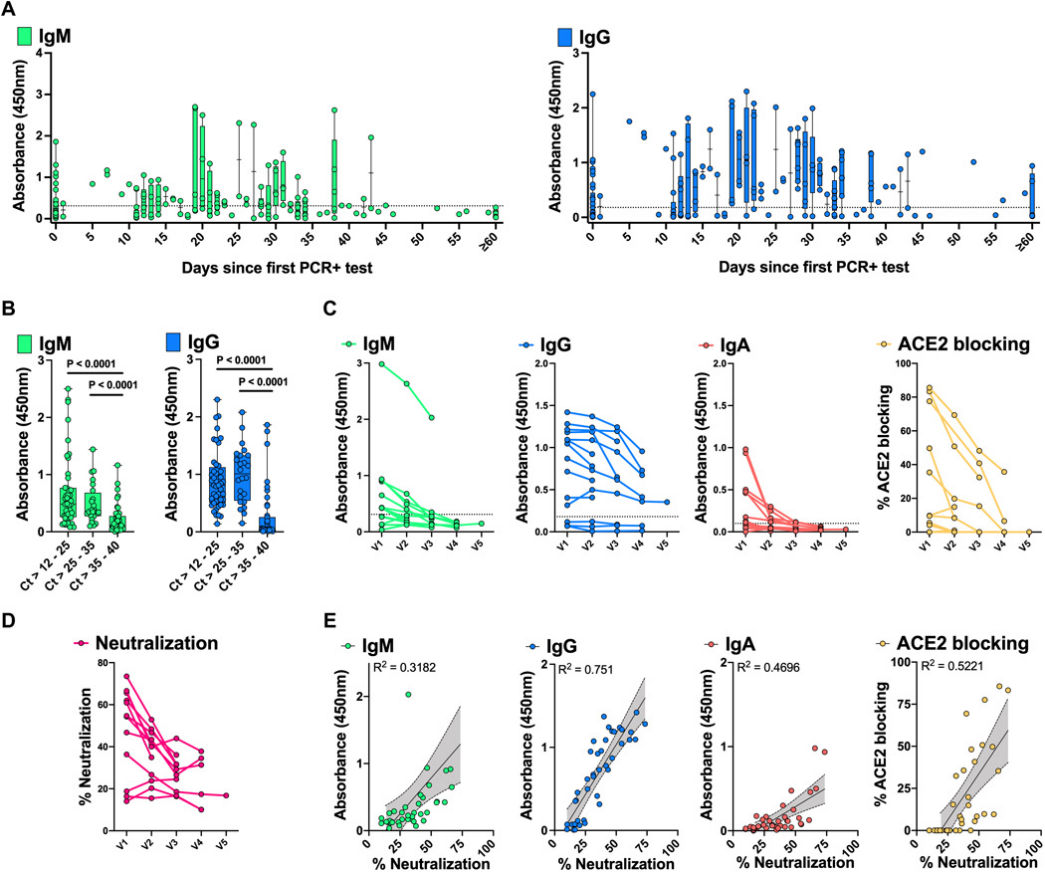
Development of anti-SARS-CoV-2 spike RBD antibody responses in SARS-CoV-2 rRT-PCR+ asymptomatic individuals and outpatients over time. 176 plasma samples from 136 SARS-CoV-2 rRT-PCR+ individuals were tested for RBD IgM and IgG. The x-axis indicates the time following the first positive rRT-PCR test (**A**). Dotted lines denote the assay cutoff for positive results. RBD IgM and IgG are shown for the latest available timepoint for subjects with low (Ct >12-25), middle (Ct >25-35) and high (Ct >35-40) SARS-CoV-2 rRT-PCR Ct at diagnosis (**B**). 45 plasma samples collected from 14 rRT-PCR+ asymptomatic individuals and outpatients sampled at monthly intervals (V1 = enrollment, V2 to V5 = months 1 to 4 post-enrollment) were tested for RBD IgM, IgG, IgA at a dilution of 1:100, as well as RBD-ACE2 blocking antibodies at a dilution of 1:10 (**C**). The 45 plasma samples were further tested for pseudoviral neutralization at a dilution of 1:1250 (**D**). Box-whisker ELISA OD_450_ and blocking/neutralization percent plots show the interquartile range as the box and the minimum and maximum values as the ends of the whiskers. Correlations between virus neutralization and RBD IgM, IgG, IgA, and RBD-ACE2 blocking are shown with superimposed simple linear regression and 95% confidence bands (grey shading) of the best-fit line (**E**). Plots show mean ELISA OD_450_ values of duplicate measurements and average percent neutralization from duplicate testing in each of two replicate experiments.

Most individuals who become infected with SARS-CoV-2 infection do not require hospitalization to recover from their illness, and a sizeable fraction (approximately 40-45%) (*29*) remain asymptomatic. We carried out further analysis of antibody responses in a larger set of samples from 136 outpatients and asymptomatic individuals who tested positive by rRT-PCR for SARS-CoV-2 RNA in nasopharyngeal swabs and had serological testing conducted in the Stanford Health Care Clinical Laboratory. We also tested an independent validation set of 45 plasma samples from an additional 14 asymptomatic or mildly ill individuals. As seen for outpatients in Fig. 2B, the asymptomatic and outpatient individuals showed relatively low titers and rapid decline of RBD IgM and IgG (Fig. 4A, table S3). The timing of infection in asymptomatic individuals is less certain than for symptomatic individuals, whose symptoms usually develop within 2 to 14 days after exposure to the virus (*30*). Plotting of serological responses of outpatients and asymptomatic individuals relative to the date of their first positive rRT-PCR test for infection nonetheless showed a similar time course to that seen for outpatients plotted as days post onset of symptoms (Fig. 2B). Notably, the amount of viral RNA detected in diagnostic nasopharyngeal swabs was correlated with the antibody titers measured in these individuals (Fig. 4B).

Plasma specimens from the independent validation cohort of asymptomatic or outpatient individuals were collected prospectively during monthly visits up to four months following recruitment (Fig. 4C, D, fig. S9). RBD IgM, IgG and IgA, RBD-ACE2 blocking and neutralization assays all showed a progressive decrease during the months sampled. Neutralizing antibodies were best correlated with RBD IgG titers, and somewhat less well with RBD-ACE2 blocking ELISA in these individuals (R^2^= 0.751 and 0.5221, respectively) (Fig. 4E).

### Outpatients and non-ICU inpatients show preferential antibody targeting of RBD and S1 compared to N

**Fig. 5:**
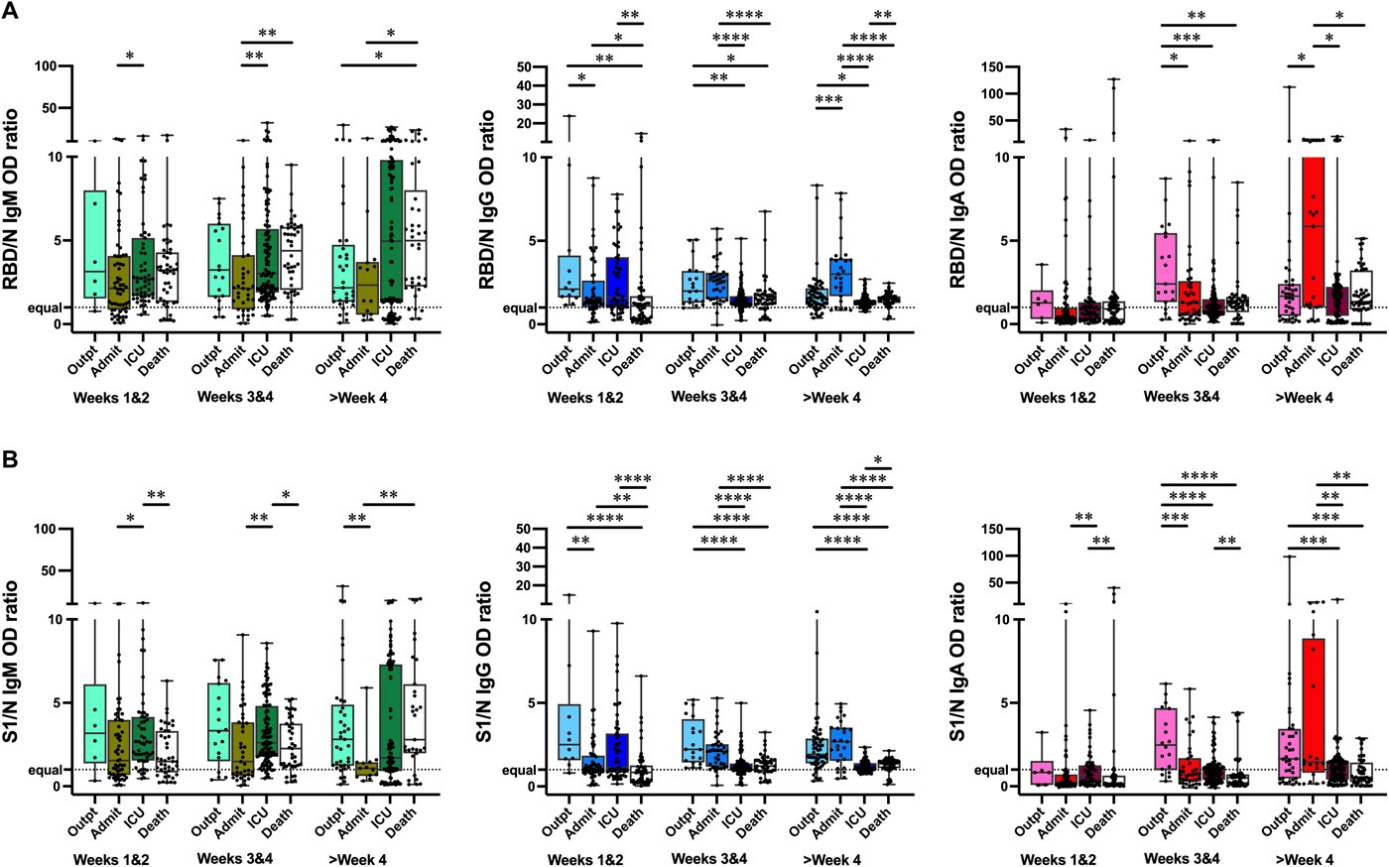
RBD/N and S1/N antibody response ratios in patients with different disease severity. Ratios of RBD/N (**A**) and anti-S1/N (**B**) IgM, IgG, and IgA ELISA OD_450_ values are shown for outpatients (Outpt), hospitalized patients who did not (Admit) or did (ICU) require ICU care, and deceased patients (Death) over time. Box-whisker ELISA OD_450_ ratio plots illustrate the interquartile range as the box and the minimum and maximum values as the ends of the whiskers. Comparisons between groups were by the two-sided Wilcoxon rank sum test. * P < 0.05, ** P < 0.01, *** P < 0.001, **** P < 0.0001. The dotted line denotes an equal ratio of antigen-specific antibodies.

It is an open question whether antibody responses in the initial weeks of SARS-CoV-2 infection have a role in modulating disease severity. Having found that patients with milder illness or asymptomatic infection had lower levels of SARS-CoV-2 antibodies to RBD, S1 and N compared to severely ill patients, we hypothesized that the relative targeting of the antibody response between antigens might be associated with different disease severity. In a recent study, serological analysis of samples from patients who died of COVID-19 compared to individuals who convalesced suggested an enrichment of an aggregate measure of spike antibodies or antibody functional activities in the convalescent (*19*). We calculated the ratios of RBD to N (Fig. 5A) and S1 to N (Fig. 5B) ELISA results for IgM, IgG and IgA for all specimens that had detectable antibodies. Notably, in weeks 1 and 2 post-onset of symptoms, outpatient IgG RBD/N and S1/N ratios were higher than those of admitted non-ICU patients, and patients who died of their illness; admitted and ICU patients also had higher RBD/N ratios compared to patients who died. In weeks 3 and 4, and after 4 weeks, outpatients and admitted patients had higher S1/N IgG ratios compared to ICU patients and those who died. IgA responses showed a similar pattern to IgG in weeks 3 and 4, and after week 4 post-onset of symptoms. The IgM ratios to these antigens was more variable and did not show a consistent relationship to disease severity.

### Inpatient SARS-CoV-2 antibody time course patterns are associated with decreases in RNAemia, but not disease outcome

**Fig. 6:**
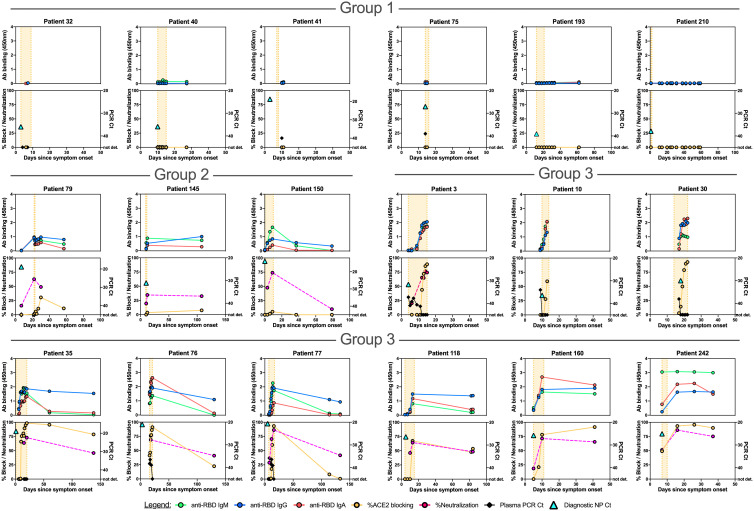
Correlations between anti-SARS-CoV-2 spike RBD antibody responses, spike pseudovirus neutralization activity, and viral RNA in individual hospitalized patients who did not require ICU care. Individual patient plots show the development of RBD IgM, IgG, and IgA antibody responses (upper panels for each patient) and RBD-ACE2 blocking antibodies (lower panels for each patient) for all available plasma sample time points. Orange shading indicates time admitted in the hospital. Representative plots for individuals with no (Group 1), up to 25% (Group 2) and greater than 25% (Group 3) RBD-ACE2 blocking activity are shown. Plots for the remaining admitted patients are shown in **fig. S10**. Viral RNAemia and pseudovirus neutralization activity are displayed in the lower panels of patients in which these were measured. The time point and Ct value for the diagnostic NP swab SARS-CoV-2 rRT PCR is shown as a turquoise triangle in the lower panel.

**Fig. 7:**
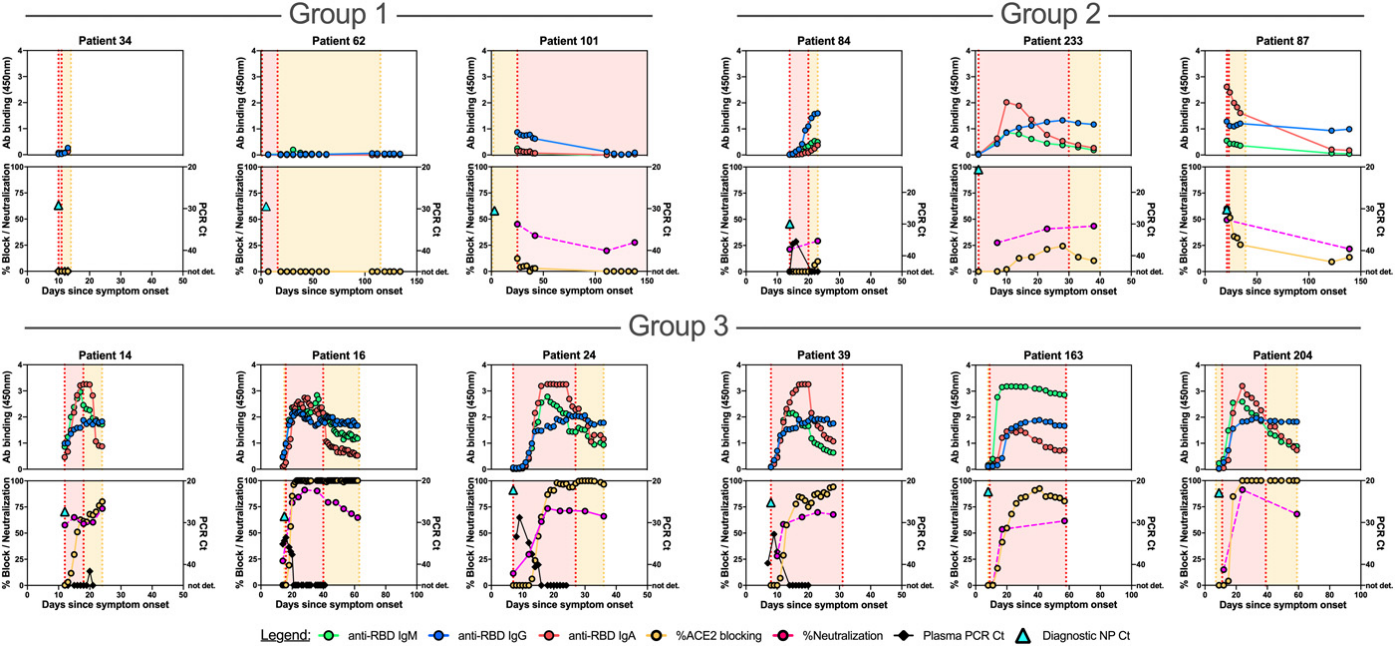
Correlations between anti-SARS-CoV-2 spike RBD antibody responses, spike pseudovirus neutralization activity, and viral RNA in individual ICU patients. Individual patient plots show the development of RBD IgM, IgG, and IgA antibody responses (upper panels for each patient) and RBD-ACE2 blocking antibodies (lower panels for each patient) for all available plasma sample time points. Orange shading indicates time admitted in the hospital, red shading represents the timeframe patients were treated in the ICU. Representative plots for individuals with no (Group 1), up to 25% (Group 2), and over 25% (Group 3) RBD-ACE2 blocking activity at the time point closest to discharge from hospital are shown here. Plots for the remaining ICU patients are shown in **fig. S11A**. The time point and Ct value for the diagnostic NP swab SARS-CoV-2 rRT PCR is shown as a turquoise triangle in the lower panel.

**Fig. 8:**
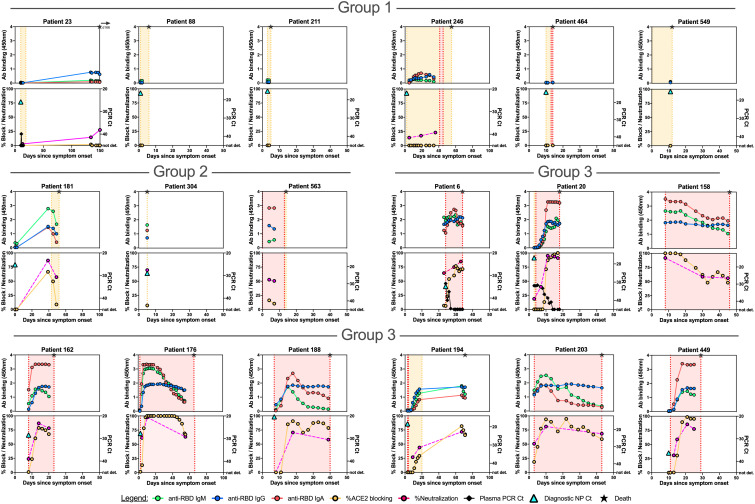
Correlations between anti-SARS-CoV-2 spike RBD antibody responses, spike pseudovirus neutralization activity, and viral RNA in individual deceased patients. Individual patient plots show the development of RBD IgM, IgG, and IgA antibody responses (upper panels for each patient) and RBD-ACE2 blocking antibodies (lower panels for each patient) for all available plasma sample time points. Orange shading indicates time admitted in the hospital, while red shading represents the timeframe patients were treated in the ICU. The time of death is indicated in each plot with a star. Representative plots for individuals with no (Group 1), up to 25% (Group 2) and over 25% (Group 3) RBD-ACE2 blocking activity are shown here. Plots for the remaining deceased patients are shown in **fig. S11B**. The time point and Ct value for the diagnostic NP swab SARS-CoV-2 rRT PCR is shown as a turquoise triangle in the lower panel.

We hypothesized that detailed examination of the time course of SARS-CoV-2 antibody responses could identify distinct patterns associated with admitted non-ICU patients (Fig. 6, fig. S10), ICU patients who recovered from their illness (Fig. 7, fig. S11A), or those who died (Fig. 8, fig. S11B). We identified three main patterns of response: at the time point closest to their discharge from hospital, or death, Group 1 individuals had SARS-CoV-2 antibody responses with no RBD IgG or RBD-ACE2 blocking activity; Group 2 individuals had up to 25% RBD-ACE2 blocking activity; and Group 3 individuals developed high levels of antibodies and over 25% RBD-ACE2 blocking activity. These patterns of response were not strongly associated with disease severity, but instead were shared across disease categories. Group 1 patients included admitted patients who recovered from their illness rapidly and were discharged before they had developed detectable antibody responses, but also included patients who recovered from their illness without antibody production after prolonged ICU stays, and patients who died of COVID-19 prior to antibody development. Similarly, Group 2 patterns with low levels of RBD-ACE2 blocking were shared across all three patient categories. In Group 3 (high levels of antibody production and >25% RBD-ACE2 blocking activity) admitted non-ICU patients differed from ICU or deceased patients in that they developed their robust antibody responses during short hospital stays prior to discharge, whereas ICU or deceased patients typically had prolonged hospital courses. In patients tested for neutralizing antibodies (13 admitted non-ICU patients, 16 ICU surviving patients, and 16 deceased patients), the results were closely correlated with IgG titers to RBD, or RBD-ACE2 blocking.

Viral RNAemia is detected in up to a third of COVID-19 patients, most often in patients with severe disease (*3*, *31*). RNAemia was evaluated in a subset of patients, and detected in 15 of 25 admitted patients, 13 of 15 ICU patients who recovered, and 2 of 2 patients who died of their disease. Reduction in RNAemia was strongly correlated with the appearance of plasma antibodies (Spearman’s correlation coefficients of -0.47 for IgM, -0.43 for IgG, and -0.44 for IgA, p<0.001 for each) (Figs. 6, 7, 8; figs. S10, S11).

## DISCUSSION

Key clinical questions in the ongoing COVID-19 pandemic are the role of antibodies in modulating disease severity during infection, the duration of individuals’ serological responses, and the extent to which patient antibody responses may be protective against reinfection. As SARS-CoV-2 vaccine candidates progress through clinical trials, comparison of vaccine-induced immune responses to those stimulated by viral infection, and those of individuals who become reinfected, will help clarify immunological correlates of protection. More comprehensive understanding of the role of antibodies in acute COVID-19 illness will guide effective use of therapeutic convalescent plasma products and recombinant antibodies targeting SARS-CoV-2. Assuming spike-targeting vaccines are shown to be safe and effective and become widely used, monitoring N-specific antibodies may be of utility in distinguishing vaccine-related antibodies from those stimulated by infection.

Here, we have analyzed serological responses to SARS-CoV-2 in 254 individuals ranging from asymptomatic individuals to ICU patients, with detailed analysis of antibody responses to SARS-CoV-2 RBD, S1 and N antigens, and functional assays testing antibody blocking of RBD-ACE2 binding or spike-pseudotyped virus neutralization. At early time points post-onset of symptoms, we saw no evidence of pre-existing antibodies that recognize SARS-CoV-2, suggesting that there is minimal cross-reactivity of SARS-CoV-2 antigens with community coronaviruses. These findings do not preclude the possibility that memory B cells stimulated by prior coronavirus exposure may be cross-reactive and form part of the response to SARS-CoV-2.

IgM, IgG and IgA antibodies specific for SARS-CoV-2 antigens typically become detectable in patients’ blood at similar median times of about 2 weeks after onset of symptoms (this study and (*32*, *33*)), although IgA timing is the most variable. As we have recently reported, large polyclonal proliferations of recently class-switched B cells expressing IgG and IgA subtypes with low antibody somatic mutation frequencies appear in the circulation around the time of seroconversion, likely including plasmablasts that contribute to the observed serological responses (*29*).

Which features of patient antibody responses were associated with disease severity? To our surprise, we found that outpatients with the mildest illness showed higher ratios of spike RBD and S1 domains compared to N antigen, beginning in the first two weeks post-onset of symptoms. These findings could suggest that early humoral immune responses focused on spike antigens help to constrain the viral infection, perhaps even at times when titers are not yet high enough to be measured in the blood. Outpatients and admitted non-ICU patients had the lowest viral loads in their nasopharyngeal swabs, but whether this is due to their antibody responses cannot be determined from this observational study. Associations of mortality in COVID-19 patients with SARS-CoV-2 viral load assessed by rRT-PCR applied to nasopharyngeal swab specimens have also been found in other studies (*34*, *35*). Our data are consistent with results reported from a panel of antibody assays applied to single time point samples from COVID-19 patients who recovered or died of their disease, which found higher values of spike-targeting responses in the convalescents (*19*). Patients with more severe illness in our study eventually raised higher antibody titers than those with milder disease, consistent with prior publications (*32*, *36*) and reports of other coronavirus infections (*21*–*23*). Patients with more severe disease also had somewhat higher viral loads than patients with milder illness, suggesting that larger initial amounts of viral antigen may contribute to their greater serological responses. Functional blocking of RBD-ACE2 interaction, and spike-pseudotyped virus neutralization by patient antibodies appeared with a similar time course to IgM, IgG, and IgA, but were most closely correlated in magnitude with IgG titers. Neutralization, RBD-ACE2 blocking, and RBD-specific IgG were all highly correlated in patients with high antibody levels, but RBD-ACE2 blocking was less sensitive than the neutralization assay, potentially because of antibodies that can neutralize by binding to non-RBD regions of the spike, or lower affinity antibodies that can neutralize in the cell culture assay but do not compete as well with binding of ACE2 under the blocking assay conditions. It is currently unclear, however, which of these assays will be the best predictor of in vivo immunological protection from SARS-CoV-2 infection or reinfection in vaccinated or previously infected individuals.

In the detailed serological time courses of the hospitalized patients in this study it was evident that the patterns of antibody responses could not fully explain patient outcomes, including death. Substantial numbers of patients recovered from their illness and were discharged from hospital before they had formed detectable antibody responses, but minimal serological responses were also seen in patients who died of COVID-19 at early times post-onset of symptoms. Similarly, individuals with moderate antibody production were seen across the full spectrum of inpatient disease severity, and many patients who died of their disease generated high levels of antibodies, RBD-ACE2 blocking activity, and neutralizing titers. Differences between individuals in other aspects of the immune response or disease course, such as production of inflammatory mediators, T cell responses, host cell and tissue vulnerability to the damage during viral infection, coagulopathy, and secondary infections, are all likely to contribute to patient outcomes.

There is an urgent need to understand how long antibody titers against the virus persist after infection, now that the pandemic has been underway for more than half a year in many countries, and initial case reports of proven reinfection by SARS-CoV-2 have begun to appear (*37*, *38*). Studies differing in their patient populations, disease severity, and serological assays, have disagreed on the duration of SARS-CoV-2 antibody responses (*14*, *16*, *32*). Our data derived from inpatients, outpatients and asymptomatic individuals, with an additional asymptomatic validation cohort, show clearly that not only IgM and IgA, but also IgG titers to RBD, S1 and N antigens, RBD-ACE2 blocking activity and spike pseudotyped viral neutralization titers all begin to decrease in patients after approximately the first month post-onset of symptoms. The decline in antibody titers is most evident in individuals who had asymptomatic infection or mild illness, who produce lower levels of antibodies at the peak of their responses. We note that reported results failing to find a decrease in SARS-CoV-2 antibodies after several months post-infection have relied on “pan-Ig” assays that cannot evaluate each isotype separately (*16*). Our data do not permit us to predict what fraction of the population will be susceptible to reinfection at a given time after their initial illness, or whether individuals will maintain sustained plateaus of lower antibody levels after an initial decrease; additional time and follow-up will be required to obtain this information. A limitation of our study is that, apart from the prospective validation cohort, the sampling time points for each patient were determined by their length of hospital stay and subsequent healthcare visits, enabling more detailed analysis of serological responses in patients with more severe illness. It is important to note that decreasing antibody levels do not necessarily indicate that all immunity will be lost. It is possible that local mucosal antibody production in the airways could help prevent or impede SARS-CoV-2-infection upon re-exposure (*39*). Even if serum antibodies wane to undetectable levels, memory B and T cells stimulated by infection could provide a faster or more effective response following future exposure. Initial reinfection reports offer some hope that SARS-CoV-2 may behave similarly to other community coronaviruses, with reinfection generally producing milder illness than the initial infection (*40*, *41*).

One implication of our finding of waning antibody levels is that seroprevalence studies may, over time, underestimate the proportion of the investigated population which has been previously infected with SARS-CoV-2. The decrease in antibodies after infection also raises the question of how long antibodies elicited by vaccination will last, and whether frequent boosting will be needed to maintain protection, assuming that safe and effective vaccines are identified. The current vaccination strategies undergoing clinical trials differ from natural infection in a variety of ways, including the method for generating or introducing viral antigens into the body, the site of exposure, and the presence of adjuvants (*42*–*45*). It is possible that some of the vaccine approaches may generate more potent and long-lasting antibodies than natural infection, in which the virus may have currently unknown mechanisms for subverting humoral immune responses. Further detailed study of the generation of memory B cell populations, short- or long-lived plasma cells, and T cell memory to SARS-CoV-2 as well as other coronaviruses should begin to clarify some of these key mechanistic points.

## MATERIALS AND METHODS

### Study design and participants

The objective of this study was to investigate correlations between humoral immune responses to SARS-CoV-2, including antibodies blocking binding of RBD to the human ACE2 receptor or neutralizing spike pseudovirus, and viral RNA loads in the nasopharynx and blood in different COVID-19 patient groups and individual patients. On March 4, 2020, the Stanford Health Care Clinical Virology Laboratory began rRT-PCR testing on nasopharyngeal specimens from suspected COVID-19 patients using a laboratory-developed SARS-CoV-2 rRT-PCR assay (*46*, *47*). For this study, we included specimens from patients with rRT-PCR-confirmed SARS-CoV-2 infection who reported with symptoms of COVID-19 to Stanford Healthcare-associated clinical sites between March 2020 and August 2020; and specimens from rRT-PCR positive outpatients and asymptomatic individuals identified between April 2020 and May 2020 through occupational health screening including rRT-PCR and serology testing for RBD IgM/G at Stanford Clinical Laboratories. The screening program that detected asymptomatic SARS-CoV-2 infected individuals was offered to all Stanford Healthcare employees on a voluntary basis, and screened employees with nasopharyngeal swab rRT-PCR testing and serology. In addition, we included a validation cohort of 14 asymptomatic and mildly ill individuals with prospective sample collection for up to four months post-enrollment. This study was approved by the Stanford University Institutional Review Board (Protocols IRB-48973 and IRB-55689).

### Sample and data collection

Venipuncture blood samples collected in sodium heparin- or K_2_EDTA-coated vacutainers were used for serology testing and rRT-PCR detection of RNAemia, respectively. After centrifugation for collection of plasma, samples were stored at -80°C.

Retrospective chart review was performed on all study participants. Collected data included age, gender, date of symptom onset, length of hospital stay, length of time admitted in the ICU, date and Ct value for the diagnostic nasopharyngeal swab rRT-PCR test result, the presence of underlying comorbidities, clinical symptoms, and mortality.

### Production of SARS-CoV and SARS-CoV-2 proteins and ACE2-mFc

The SARS-CoV and SARS-CoV-2 RBD proteins were expressed in Expi293F cells and purified using Nickel-NTA resin and size exclusion chromatography. The SARS-CoV construct (RBD-His_pTT5, GenBank AAP13441.1) was synthesized commercially by Twist Bioscience (San Francisco, CA); the SARS-CoV-2 construct (RBD-His_pCAGGS, GenBank MN908947.3) was kindly provided by Dr. Florian Krammer (*48*). SARS-CoV-2 S1 (spike residues 1-682) and ACE2-mFc, expressed in HEK293 cells, and the N protein, expressed in *E. coli* were produced by the ATUM contract research organization. Soluble human ACE2 fused to a mouse Fc tag was constructed by synthesizing a gene encoding ACE2 (residues 1-615) joined by a (G_4_S)x2 linker to mouse IgG2a Fc, and placed under control of a CMV promoter by cloning into a mammalian expression plasmid.

### ELISA to detect anti-SARS-CoV-2 Spike RBD antibodies in plasma samples

The ELISA procedure in this study was modified from a protocol published by Stadlbauer (*48*).

96-well Corning Costar high binding plates (catalog no. 9018, Thermo Fisher) were coated with SARS-CoV RBD, SARS-CoV-2 RBD, S1, or N protein in phosphate-buffered saline (PBS) at a concentration of 0.1 μg per well (0.025 μg per well for the nucleocapsid IgG assay) overnight at 4°C. On the next day, wells were washed 3x with PBS-0.1% Tween 20 (PBS-T) and blocked with PBS-T containing 3% non-fat milk powder for 1 hour at room temperature (RT). Wells were then incubated with plasma samples from COVID-19 patients at a dilution of 1:100 in PBS-T containing 1% non-fat milk for 1 hour at 37°C. Two negative and two positive plasma pool wells and two blank wells incubated with PBS-T containing 1% non-fat milk powder were included on each plate. After washing 3x with PBS-T, horseradish peroxidase conjugated goat anti-human IgG (γ-chain specific, catalog no. 62-8420, Thermo Fisher, 1:6,000 dilution), IgM (μ-chain specific, catalog no. A6907, Sigma, 1:6,000 dilution), or IgA (α-chain specific, catalog no. P0216, Agilent, 1:5,000 dilution) in PBS-T containing 1% non-fat milk was added and incubated for 1 hour at RT. Wells were washed 3x with PBS-T and dried by vigorous tapping of plates on paper towels. 3,3′,5,5′-Tetramethylbenzidine (TMB) substrate solution was added and the reaction was stopped after 12 min by addition of 0.16 M sulfuric acid. The optical density (OD) at 450 nanometers was measured with an EMax Plus microplate reader (Molecular Devices, San Jose, CA); values for blank wells were subtracted from values obtained for plasma samples. The cutoff value for seroconversion was calculated by adding 3 standard deviations to mean ELISA ODs of 94 historical negative control samples from healthy blood donors (collected before the pandemic for an unrelated seroprevalence study) obtained by testing the samples in all protein/isotype assays (table S4). Additional details for the manual and clinical lab instrument ELISA assay setup are provided in figs. S12 and S13.

### Competition ELISA to detect antibodies blocking binding of ACE2 to RBD

96-well Corning Costar high binding plates (Thermo Fisher: cat. 9018) were coated with SARS-CoV-2 spike RBD protein in PBS at a concentration of 0.1 μg per well overnight at 4°C. All competition ELISA steps were carried out on the next day at RT. Wells were washed 3x with PBS-T and blocked with PBS-T containing 3% non-fat milk powder for 1 hour. Wells were then incubated with plasma samples from COVID-19 patients at a dilution of 1:10 in PBS-T containing 1% non-fat milk for 1 hour. Two quality controls (Access SARS-CoV-2 IgG QC, QC1-QC2, 2 levels, catalog no. C58964, Beckman Coulter) and two blank wells incubated with PBS-T containing 1% non-fat milk were included on each plate. ACE2-mFc diluted to 0.5 μg/ml in 1% non-fat milk powder was added without washing steps and incubated for an additional 45 min. After washing 3x with PBS-T, horseradish peroxidase conjugated goat anti-mouse IgG (Fc specific, catalog no. 31439, Invitrogen, 1:10’000 dilution) in PBS-T containing 1% non-fat milk was added and incubated for 45 min. Wells were washed 3x with PBS-T and dried by vigorous tapping of plates on paper towels. TMB substrate solution was added and the reaction was stopped after 12 min by addition of 0.16 M sulfuric acid. The OD at 450 nanometers was measured with an EMax Plus microplate reader (Molecular Devices, San Jose, CA). OD values were converted to percentage of blocking using the following formula: 100*(1-(sample OD - 0.2)/(QC1 OD – 0.2)), taking into account the background noise of the assay of 0.2 as determined after testing pre-pandemic control plasma samples. Additional details for competition ELISA assay setup and optimization are provided in figs. S14 and S15.

### Pseudotyped lentiviral neutralization assay

SARS-CoV-2 pseudotyped lentivirus assays were performed as described previously. Briefly, spike-pseudotyped lentivirus was produced in HEK293T cells as described in (*49*) using a 5-plasmid system described in (*27*). For viral neutralization assays, ACE2/HeLa cells (*28*) were plated in 96-well tissue culture plates one day prior to infection. Prior to neutralization assays, patient plasma samples were heat inactivated for 1 hour at 56°C. Plasma samples and virus were incubated with the cells at 37°C for ~48 hours. After incubation, cells were lysed with BriteLite assay readout solution (Perkin Elmer) and luminescence values were obtained with a BioTek plate reader. Single-dilution point neutralization assays were performed in technical duplicate in two different experimental replicates. Percent neutralization was determined by normalizing raw luciferase values to 0% infectivity (average from cells only wells) and 100% infectivity (average from virus only wells) in GraphPad Prism 8.4.1. Plasma dilution series shown in Figure S9 were performed in technical duplicate; normalized % infectivity values were fit with a three-parameter non-linear regression inhibitor curve in GraphPad Prism 8.4.1 and fits were constrained to have a value of 0% at the bottom of the fit.

### Real-time PCR to detect SARS-CoV-2 RNA in plasma

A volume of 400 μL of EDTA-anticoagulated plasma was extracted by Qiagen EZ1 Virus Mini Kit v2.0 (Qiagen Germantown, MD). Molecular testing for the presence of SARS-CoV-2 RNA in plasma was performed with a modification of a published rRT-PCR assay targeting the envelope (*E*) gene (*46*, *47*). The standard Ct values of positive tests with this assay range from Ct <20 to 45 cycles. Testing of plasma samples with a Ct value of 40 or greater was repeated to ensure reproducibility of the positive result. As viral culture was not performed as part of this study, presence of SARS-CoV-2 in tested plasma was defined as RNAemia.

### Statistics

GraphPad Prism version 8.0 (GraphPad Software, San Diego, California, USA) software was used to visualize ELISA data, analyze for differences in antibody responses between disease categories by Wilcoxon rank sum testing, and carry out linear regression of pseudotyped virus neutralization, RBD-ACE2 blocking and antibody ELISA results. The goodness of fit for linear regression analyses was reported as the coefficient of determination, R^2^. Locally estimated scatterplot smoothing for the development and decrease of antibody responses over time was performed using the loess method in the R statistical package version 3.6.1 (*50*). Correlation between antibody OD_450_ values, RNAemia, and ACE2-RBD blocking assay OD_450_ values were calculated as Spearman correlations with the R cor function.

## Supplementary Materials

immunology.sciencemag.org/cgi/content/full/5/54/eabe0240/DC1 Fig. S1. Study design and participant overview. Fig. S2. SARS-CoV-2 rRT-PCR cycle threshold (Ct) values for diagnostic nasopharyngeal (NP) swabs. Fig. S3. Distribution and trajectories of antibody responses to SARS-CoV-2 in inpatients over time. Fig. S4. Distribution and trajectories of antibody responses to SARS-CoV-2 in outpatients over time. Fig. S5. Development of anti-SARS-CoV-2 spike S1 antibody responses in COVID-19 patients. Fig. S6. Development of anti-SARS-CoV-2 N antibody responses in COVID-19 patients. Fig. S7. Comparison of antibody responses against SARS-CoV-2 antigens among ICU patients who survived or died. Fig. S8. COVID-19 inpatients develop anti-SARS-CoV/SARS-CoV-2 spike RBD cross-reactive IgG responses. Fig. S9. Pseudoviral neutralization activity in longitudinal samples from the validation cohort. Fig. S10. Correlations between anti-SARS-CoV-2 spike RBD antibody responses, pseudoviral neutralization activity, and viral RNA in individual hospitalized patients. Fig. S11. Correlations between anti-SARS-CoV-2 spike RBD antibody responses, pseudoviral neutralization activity, and viral RNA in individual ICU and deceased patients. Fig. S12. Checkerboard titration for serological RBD ELISA. Fig. S13. Validation of the Stanford Health Care Clinical Laboratory anti-RBD IgM/G ELISA. Fig. S14. Checkerboard titration for receptor blocking ELISA. Fig. S15. RBD-ACE2 blocking ELISA optimization. Table S1. Demographic and clinical characteristics of outpatients and asymptomatic individuals with and without plasma availability. Table S2. Inpatient seropositivity. Table S3. Outpatient and asymptomatic individuals’ seropositivity. Table S4. Determination of SARS-CoV-2 assay cutoff values and specificity. Table S5. Raw data file (Excel spreadsheet).

## References

[1] P. Zhou, X.-L. Yang, X.-G. Wang, B. Hu, L. Zhang, W. Zhang, H.-R. Si, Y. Zhu, B. Li, C.-L. Huang, H.-D. Chen, J. Chen, Y. Luo, H. Guo, R.-D. Jiang, M.-Q. Liu, Y. Chen, X.-R. Shen, X. Wang, X.-S. Zheng, K. Zhao, Q.-J. Chen, F. Deng, L.-L. Liu, B. Yan, F.-X. Zhan, Y.-Y. Wang, G.-F. Xiao, Z.-L. Shi, A pneumonia outbreak associated with a new coronavirus of probable bat origin. Nature 579, 270–273 (2020). 10.1038/s41586-020-2012-7 32015507 PMC7095418

[2] Z. Wu, J. M. McGoogan, Characteristics of and Important Lessons From the Coronavirus Disease 2019 (COVID-19) Outbreak in China: Summary of a Report of 72 314 Cases From the Chinese Center for Disease Control and Prevention. JAMA 323, 1239–1242 (2020). 10.1001/jama.2020.2648 32091533

[3] C. Huang, Y. Wang, X. Li, L. Ren, J. Zhao, Y. Hu, L. Zhang, G. Fan, J. Xu, X. Gu, Z. Cheng, T. Yu, J. Xia, Y. Wei, W. Wu, X. Xie, W. Yin, H. Li, M. Liu, Y. Xiao, H. Gao, L. Guo, J. Xie, G. Wang, R. Jiang, Z. Gao, Q. Jin, J. Wang, B. Cao, Clinical features of patients infected with 2019 novel coronavirus in Wuhan, China. Lancet 395, 497–506 (2020). 10.1016/S0140-6736(20)30183-5 31986264 PMC7159299

[4] N. Chen, M. Zhou, X. Dong, J. Qu, F. Gong, Y. Han, Y. Qiu, J. Wang, Y. Liu, Y. Wei, J. Xia, T. Yu, X. Zhang, L. Zhang, Epidemiological and clinical characteristics of 99 cases of 2019 novel coronavirus pneumonia in Wuhan, China: A descriptive study. Lancet 395, 507–513 (2020). 10.1016/S0140-6736(20)30211-7 32007143 PMC7135076

[5] F. Zhou, T. Yu, R. Du, G. Fan, Y. Liu, Z. Liu, J. Xiang, Y. Wang, B. Song, X. Gu, L. Guan, Y. Wei, H. Li, X. Wu, J. Xu, S. Tu, Y. Zhang, H. Chen, B. Cao, Clinical course and risk factors for mortality of adult inpatients with COVID-19 in Wuhan, China: A retrospective cohort study. Lancet 395, 1054–1062 (2020). 10.1016/S0140-6736(20)30566-3 32171076 PMC7270627

[6] S. Richardson, J. S. Hirsch, M. Narasimhan, J. M. Crawford, T. McGinn, K. W. Davidson, D. P. Barnaby, L. B. Becker, J. D. Chelico, S. L. Cohen, J. Cookingham, K. Coppa, M. A. Diefenbach, A. J. Dominello, J. Duer-Hefele, L. Falzon, J. Gitlin, N. Hajizadeh, T. G. Harvin, D. A. Hirschwerk, E. J. Kim, Z. M. Kozel, L. M. Marrast, J. N. Mogavero, G. A. Osorio, M. Qiu, T. P. Zanos; the Northwell COVID-19 Research Consortium, Presenting Characteristics, Comorbidities, and Outcomes Among 5700 Patients Hospitalized With COVID-19 in the New York City Area. JAMA 323, 2052–2059 (2020). 10.1001/jama.2020.6775 32320003 PMC7177629

[7] T. Chen, D. Wu, H. Chen, W. Yan, D. Yang, G. Chen, K. Ma, D. Xu, H. Yu, H. Wang, T. Wang, W. Guo, J. Chen, C. Ding, X. Zhang, J. Huang, M. Han, S. Li, X. Luo, J. Zhao, Q. Ning, Clinical characteristics of 113 deceased patients with coronavirus disease 2019: Retrospective study. BMJ 368, m1091 (2020). 10.1136/bmj.m1091 32217556 PMC7190011

[8] J. Perez-Saez, S. A. Lauer, L. Kaiser, S. Regard, E. Delaporte, I. Guessous, S. Stringhini, A. S. Azman; Serocov-POP Study Group, Serology-informed estimates of SARS-CoV-2 infection fatality risk in Geneva, Switzerland. Lancet Infect. Dis. 0, S1473-3099(20)30584-3 (2020). 10.1016/S1473-3099(20)30584-3 32679085 PMC7833057

[9] A. E. Gorbalenya, S. C. Baker, R. S. Baric, R. J. de Groot, C. Drosten, A. A. Gulyaeva, B. L. Haagmans, C. Lauber, A. M. Leontovich, B. W. Neuman, D. Penzar, S. Perlman, L. L. M. Poon, D. V. Samborskiy, I. A. Sidorov, I. Sola, J. Ziebuhr; Coronaviridae Study Group of the International Committee on Taxonomy of Viruses, The species Severe acute respiratory syndrome-related coronavirus: Classifying 2019-nCoV and naming it SARS-CoV-2. Nat. Microbiol. 5, 536–544 (2020). 10.1038/s41564-020-0695-z 32123347 PMC7095448

[10] W. Tai, L. He, X. Zhang, J. Pu, D. Voronin, S. Jiang, Y. Zhou, L. Du, Characterization of the receptor-binding domain (RBD) of 2019 novel coronavirus: Implication for development of RBD protein as a viral attachment inhibitor and vaccine. Cell. Mol. Immunol. 17, 613–620 (2020). 10.1038/s41423-020-0400-4 32203189 PMC7091888

[11] F. Li, Structure, Function, and Evolution of Coronavirus Spike Proteins. Annu. Rev. Virol. 3, 237–261 (2016). 10.1146/annurev-virology-110615-042301 27578435 PMC5457962

[12] B. Ju, Q. Zhang, J. Ge, R. Wang, J. Sun, X. Ge, J. Yu, S. Shan, B. Zhou, S. Song, X. Tang, J. Yu, J. Lan, J. Yuan, H. Wang, J. Zhao, S. Zhang, Y. Wang, X. Shi, L. Liu, J. Zhao, X. Wang, Z. Zhang, L. Zhang, Human neutralizing antibodies elicited by SARS-CoV-2 infection. Nature 584, 115–119 (2020). 10.1038/s41586-020-2380-z 32454513

[13] M. Ho, Perspectives on the development of neutralizing antibodies against SARS-CoV-2. Antibody Ther 3, 109–114 (2020). 10.1093/abt/tbaa009 32566896 PMC7291920

[14] F. J. Ibarrondo, J. A. Fulcher, D. Goodman-Meza, J. Elliott, C. Hofmann, M. A. Hausner, K. G. Ferbas, N. H. Tobin, G. M. Aldrovandi, O. O. Yang, Rapid Decay of Anti-SARS-CoV-2 Antibodies in Persons with Mild Covid-19. N. Engl. J. Med. 383, 1085–1087 (2020). 10.1056/NEJMc2025179 32706954 PMC7397184

[15] Q.-X. Long, X.-J. Tang, Q.-L. Shi, Q. Li, H.-J. Deng, J. Yuan, J.-L. Hu, W. Xu, Y. Zhang, F.-J. Lv, K. Su, F. Zhang, J. Gong, B. Wu, X.-M. Liu, J.-J. Li, J.-F. Qiu, J. Chen, A.-L. Huang, Clinical and immunological assessment of asymptomatic SARS-CoV-2 infections. Nat. Med. 26, 1200–1204 (2020). 10.1038/s41591-020-0965-6 32555424

[16] D. F. Gudbjartsson, G. L. Norddahl, P. Melsted, K. Gunnarsdottir, H. Holm, E. Eythorsson, A. O. Arnthorsson, D. Helgason, K. Bjarnadottir, R. F. Ingvarsson, B. Thorsteinsdottir, S. Kristjansdottir, K. Birgisdottir, A. M. Kristinsdottir, M. I. Sigurdsson, G. A. Arnadottir, E. V. Ivarsdottir, M. Andresdottir, F. Jonsson, A. B. Agustsdottir, J. Berglund, B. Eiriksdottir, R. Fridriksdottir, E. E. Gardarsdottir, M. Gottfredsson, O. S. Gretarsdottir, S. Gudmundsdottir, K. R. Gudmundsson, T. R. Gunnarsdottir, A. Gylfason, A. Helgason, B. O. Jensson, A. Jonasdottir, H. Jonsson, T. Kristjansson, K. G. Kristinsson, D. N. Magnusdottir, O. T. Magnusson, L. B. Olafsdottir, S. Rognvaldsson, L. le Roux, G. Sigmundsdottir, A. Sigurdsson, G. Sveinbjornsson, K. E. Sveinsdottir, M. Sveinsdottir, E. A. Thorarensen, B. Thorbjornsson, M. Thordardottir, J. Saemundsdottir, S. H. Kristjansson, K. S. Josefsdottir, G. Masson, G. Georgsson, M. Kristjansson, A. Moller, R. Palsson, T. Gudnason, U. Thorsteinsdottir, I. Jonsdottir, P. Sulem, K. Stefansson, Humoral Immune Response to SARS-CoV-2 in Iceland. N. Engl. J. Med. 383, 1724–1734 (2020). 10.1056/NEJMoa2026116 32871063 PMC7494247

[17] Y. Wang, L. Zhang, L. Sang, F. Ye, S. Ruan, B. Zhong, T. Song, A. N. Alshukairi, R. Chen, Z. Zhang, M. Gan, A. Zhu, Y. Huang, L. Luo, C. K. P. Mok, M. M. Al Gethamy, H. Tan, Z. Li, X. Huang, F. Li, J. Sun, Y. Zhang, L. Wen, Y. Li, Z. Chen, Z. Zhuang, J. Zhuo, C. Chen, L. Kuang, J. Wang, H. Lv, Y. Jiang, M. Li, Y. Lin, Y. Deng, L. Tang, J. Liang, J. Huang, S. Perlman, N. Zhong, J. Zhao, J. S. Malik Peiris, Y. Li, J. Zhao, Kinetics of viral load and antibody response in relation to COVID-19 severity. J. Clin. Invest. 130, 5235–5244 (2020). 10.1172/JCI138759 32634129 PMC7524490

[18] B. Isho, K. T. Abe, M. Zuo, A. J. Jamal, B. Rathod, J. H. Wang, Z. Li, G. Chao, O. L. Rojas, Y. M. Bang, A. Pu, N. Christie-Holmes, C. Gervais, D. Ceccarelli, P. Samavarchi-Tehrani, F. Guvenc, P. Budylowski, A. Li, A. Paterson, F. Y. Yue, L. M. Marin, L. Caldwell, J. L. Wrana, K. Colwill, F. Sicheri, S. Mubareka, S. D. Gray-Owen, S. J. Drews, W. L. Siqueira, M. Barrios-Rodiles, M. Ostrowski, J. M. Rini, Y. Durocher, A. J. McGeer, J. L. Gommerman, A.-C. Gingras, Persistence of serum and saliva antibody responses to SARS-CoV-2 spike antigens in COVID-19 patients. Sci. Immunol. 5, eabe5511 (2020). 10.1126/sciimmunol.abe5511 33033173 PMC8050884

[19] C. Atyeo, S. Fischinger, T. Zohar, M. D. Slein, J. Burke, C. Loos, D. J. McCulloch, K. L. Newman, C. Wolf, J. Yu, K. Shuey, J. Feldman, B. M. Hauser, T. Caradonna, A. G. Schmidt, T. J. Suscovich, C. Linde, Y. Cai, D. Barouch, E. T. Ryan, R. C. Charles, D. Lauffenburger, H. Chu, G. Alter, Distinct Early Serological Signatures Track with SARS-CoV-2 Survival. Immunity 53, 524–532.e4 (2020). 10.1016/j.immuni.2020.07.020 32783920 PMC7392190

[20] J. Yu, L. H. Tostanoski, L. Peter, N. B. Mercado, K. McMahan, S. H. Mahrokhian, J. P. Nkolola, J. Liu, Z. Li, A. Chandrashekar, D. R. Martinez, C. Loos, C. Atyeo, S. Fischinger, J. S. Burke, M. D. Slein, Y. Chen, A. Zuiani, F. J. N. Lelis, M. Travers, S. Habibi, L. Pessaint, A. Van Ry, K. Blade, R. Brown, A. Cook, B. Finneyfrock, A. Dodson, E. Teow, J. Velasco, R. Zahn, F. Wegmann, E. A. Bondzie, G. Dagotto, M. S. Gebre, X. He, C. Jacob-Dolan, M. Kirilova, N. Kordana, Z. Lin, L. F. Maxfield, F. Nampanya, R. Nityanandam, J. D. Ventura, H. Wan, Y. Cai, B. Chen, A. G. Schmidt, D. R. Wesemann, R. S. Baric, G. Alter, H. Andersen, M. G. Lewis, D. H. Barouch, DNA vaccine protection against SARS-CoV-2 in rhesus macaques. Science 369, 806–811 (2020). 10.1126/science.abc6284 32434945 PMC7243363

[21] N. M. A. Okba, V. S. Raj, I. Widjaja, C. H. GeurtsvanKessel, E. de Bruin, F. D. Chandler, W. B. Park, N.-J. Kim, E. A. B. A. Farag, M. Al-Hajri, B.-J. Bosch, M. D. Oh, M. P. G. Koopmans, C. B. E. M. Reusken, B. L. Haagmans, Sensitive and Specific Detection of Low-Level Antibody Responses in Mild Middle East Respiratory Syndrome Coronavirus Infections. Emerg. Infect. Dis. 25, 1868–1877 (2019). 10.3201/eid2510.190051 31423970 PMC6759241

[22] A. N. Alshukairi, I. Khalid, W. A. Ahmed, A. M. Dada, D. T. Bayumi, L. S. Malic, S. Althawadi, K. Ignacio, H. S. Alsalmi, H. M. Al-Abdely, G. Y. Wali, I. A. Qushmaq, B. M. Alraddadi, S. Perlman, Antibody Response and Disease Severity in Healthcare Worker MERS Survivors. Emerg. Infect. Dis. 22, (2016). 10.3201/eid2206.160010 27192543 PMC4880093

[23] C. Drosten, B. Meyer, M. A. Müller, V. M. Corman, M. Al-Masri, R. Hossain, H. Madani, A. Sieberg, B. J. Bosch, E. Lattwein, R. F. Alhakeem, A. M. Assiri, W. Hajomar, A. M. Albarrak, J. A. Al-Tawfiq, A. I. Zumla, Z. A. Memish, Transmission of MERS-coronavirus in household contacts. N. Engl. J. Med. 371, 828–835 (2014). 10.1056/NEJMoa1405858 25162889

[24] X. Chen, Z. Pan, S. Yue, F. Yu, J. Zhang, Y. Yang, R. Li, B. Liu, X. Yang, L. Gao, Z. Li, Y. Lin, Q. Huang, L. Xu, J. Tang, L. Hu, J. Zhao, P. Liu, G. Zhang, Y. Chen, K. Deng, L. Ye, Disease severity dictates SARS-CoV-2-specific neutralizing antibody responses in COVID-19. Signal Transduct. Target. Ther. 5, 180 (2020). 10.1038/s41392-020-00301-9 32879307 PMC7464057

[25] X. Tian, C. Li, A. Huang, S. Xia, S. Lu, Z. Shi, L. Lu, S. Jiang, Z. Yang, Y. Wu, T. Ying, Potent binding of 2019 novel coronavirus spike protein by a SARS coronavirus-specific human monoclonal antibody. Emerg. Microbes Infect. 9, 382–385 (2020). 10.1080/22221751.2020.1729069 32065055 PMC7048180

[26] D. Wrapp, N. Wang, K. S. Corbett, J. A. Goldsmith, C.-L. Hsieh, O. Abiona, B. S. Graham, J. S. McLellan, Cryo-EM structure of the 2019-nCoV spike in the prefusion conformation. Science 367, 1260–1263 (2020). 10.1126/science.abb2507 32075877 PMC7164637

[27] K. H. D. Crawford, R. Eguia, A. S. Dingens, A. N. Loes, K. D. Malone, C. R. Wolf, H. Y. Chu, M. A. Tortorici, D. Veesler, M. Murphy, D. Pettie, N. P. King, A. B. Balazs, J. D. Bloom, Protocol and Reagents for Pseudotyping Lentiviral Particles with SARS-CoV-2 Spike Protein for Neutralization Assays. Viruses 12, 513 (2020). 10.3390/v12050513 32384820 PMC7291041

[28] T. F. Rogers, F. Zhao, D. Huang, N. Beutler, A. Burns, W. T. He, O. Limbo, C. Smith, G. Song, J. Woehl, L. Yang, R. K. Abbott, S. Callaghan, E. Garcia, J. Hurtado, M. Parren, L. Peng, S. Ramirez, J. Ricketts, M. J. Ricciardi, S. A. Rawlings, N. C. Wu, M. Yuan, D. M. Smith, D. Nemazee, J. R. Teijaro, J. E. Voss, I. A. Wilson, R. Andrabi, B. Briney, E. Landais, D. Sok, J. G. Jardine, D. R. Burton, Isolation of potent SARS-CoV-2 neutralizing antibodies and protection from disease in a small animal model. Science 369, 956–963 (2020). 10.1126/science.abc7520 32540903 PMC7299280

[29] D. P. Oran, E. J. Topol, Prevalence of Asymptomatic SARS-CoV-2 Infection : A Narrative Review. Ann. Intern. Med. 173, 362–367 (2020). 10.7326/M20-3012 32491919 PMC7281624

[30] CDC, Coronavirus Disease 2019 (COVID-19) – Symptoms*Centers for Disease Control and Prevention* (2020) (available at https://www.cdc.gov/coronavirus/2019-ncov/symptoms-testing/symptoms.html).

[31] C. A. Hogan, B. A. Stevens, M. K. Sahoo, C. Huang, N. Garamani, S. Gombar, F. Yamamoto, K. Murugesan, J. Kurzer, J. Zehnder, B. A. Pinsky, High Frequency of SARS-CoV-2 RNAemia and Association With Severe Disease. Clin. Infect. Dis. ciaa1054 (2020). 10.1093/cid/ciaa1054 32965474 PMC7543277

[32] Q.-X. Long, B.-Z. Liu, H.-J. Deng, G.-C. Wu, K. Deng, Y.-K. Chen, P. Liao, J.-F. Qiu, Y. Lin, X.-F. Cai, D.-Q. Wang, Y. Hu, J.-H. Ren, N. Tang, Y.-Y. Xu, L.-H. Yu, Z. Mo, F. Gong, X.-L. Zhang, W.-G. Tian, L. Hu, X.-X. Zhang, J.-L. Xiang, H.-X. Du, H.-W. Liu, C.-H. Lang, X.-H. Luo, S.-B. Wu, X.-P. Cui, Z. Zhou, M.-M. Zhu, J. Wang, C.-J. Xue, X.-F. Li, L. Wang, Z.-J. Li, K. Wang, C.-C. Niu, Q.-J. Yang, X.-J. Tang, Y. Zhang, X.-M. Liu, J.-J. Li, D.-C. Zhang, F. Zhang, P. Liu, J. Yuan, Q. Li, J.-L. Hu, J. Chen, A.-L. Huang, Antibody responses to SARS-CoV-2 in patients with COVID-19. Nat. Med. 26, 845–848 (2020). 10.1038/s41591-020-0897-1 32350462

[33] A. S. Iyer, F. K. Jones, A. Nodoushani, M. Kelly, M. Becker, D. Slater, R. Mills, E. Teng, M. Kamruzzaman, W. F. Garcia-Beltran, M. Astudillo, D. Yang, T. E. Miller, E. Oliver, S. Fischinger, C. Atyeo, A. J. Iafrate, S. B. Calderwood, S. A. Lauer, J. Yu, Z. Li, J. Feldman, B. M. Hauser, T. M. Caradonna, J. A. Branda, S. E. Turbett, R. C. LaRocque, G. Mellon, D. H. Barouch, A. G. Schmidt, A. S. Azman, G. Alter, E. T. Ryan, J. B. Harris, R. C. Charles, Persistence and decay of human antibody responses to the receptor binding domain of SARS-CoV-2 spike protein in COVID-19 patients. Sci. Immunol. 5, eabe0367 (2020). 10.1126/sciimmunol.abe0367 33033172 PMC7857394

[34] L. F. Westblade, G. Brar, L. C. Pinheiro, D. Paidoussis, M. Rajan, P. Martin, P. Goyal, J. L. Sepulveda, L. Zhang, G. George, D. Liu, S. Whittier, M. Plate, C. B. Small, J. H. Rand, M. M. Cushing, T. J. Walsh, J. Cooke, M. M. Safford, M. Loda, M. J. Satlin, SARS-CoV-2 Viral Load Predicts Mortality in Patients with and without Cancer Who Are Hospitalized with COVID-19. Cancer Cell 38, 661–671.e2 (2020). 10.1016/j.ccell.2020.09.007 32997958 PMC7492074

[35] R. Magleby, L. F. Westblade, A. Trzebucki, M. S. Simon, M. Rajan, J. Park, P. Goyal, M. M. Safford, M. J. Satlin, Impact of SARS-CoV-2 Viral Load on Risk of Intubation and Mortality Among Hospitalized Patients with Coronavirus Disease 2019. Nephrol. Dial. Transplant. ciaa851 (2020). 10.1093/cid/ciaa851 32603425 PMC7337625

[36] A. Casadevall, L. A. Pirofski, The convalescent sera option for containing COVID-19. J. Clin. Invest. 130, 1545–1548 (2020). 10.1172/JCI138003 32167489 PMC7108922

[37] J. D. Goldman, K. Wang, K. Roltgen, S. C. A. Nielsen, J. C. Roach, S. N. Naccache, F. Yang, O. F. Wirz, K. E. Yost, J.-Y. Lee, K. Chun, T. Wrin, C. J. Petropoulos, I. Lee, S. Fallen, P. M. Manner, J. A. Wallick, H. A. Algren, K. M. Murray, Y. Su, J. Hadlock, J. Jeharajah, W. R. Berrington, G. P. Pappas, S. T. Nyatsatsang, A. L. Greninger, A. T. Satpathy, J. S. Pauk, S. D. Boyd, J. R. Heath, Reinfection with SARS-CoV-2 and Failure of Humoral Immunity: A case report. medRxiv 2020.09.22.20192443 (2020). 10.1101/2020.09.22.20192443 36679852 PMC9861578

[38] K. K.-W. To, I. F.-N. Hung, J. D. Ip, A. W.-H. Chu, W.-M. Chan, A. R. Tam, C. H.-Y. Fong, S. Yuan, H.-W. Tsoi, A. C.-K. Ng, L. L.-Y. Lee, P. Wan, E. Tso, W.-K. To, D. Tsang, K.-H. Chan, J.-D. Huang, K.-H. Kok, V. C.-C. Cheng, K.-Y. Yuen, COVID-19 re-infection by a phylogenetically distinct SARS-coronavirus-2 strain confirmed by whole genome sequencing. Nephrol. Dial. Transplant. ciaa1275 (2020). 10.1093/cid/ciaa1275 32840608 PMC7499500

[39] C. Cervia, J. Nilsson, Y. Zurbuchen, A. Valaperti, J. Schreiner, A. Wolfensberger, M. E. Raeber, S. Adamo, S. Weigang, M. Emmenegger, S. Hasler, P. P. Bosshard, E. De Cecco, E. Bächli, A. Rudiger, M. Stüssi-Helbling, L. C. Huber, A. S. Zinkernagel, D. J. Schaer, A. Aguzzi, G. Kochs, U. Held, E. Probst-Müller, S. K. Rampini, O. Boyman, Systemic and mucosal antibody responses specific to SARS-CoV-2 during mild versus severe COVID-19. J. Allergy Clin. Immunol. S0091-6749(20)31623-7 (2020). 10.1016/j.jaci.2020.10.040 33221383 PMC7677074

[40] A. W. D. Edridge, J. Kaczorowska, A. C. R. Hoste, M. Bakker, M. Klein, K. Loens, M. F. Jebbink, A. Matser, C. M. Kinsella, P. Rueda, M. Ieven, H. Goossens, M. Prins, P. Sastre, M. Deijs, L. van der Hoek, Seasonal coronavirus protective immunity is short-lasting. Nat. Med. 26, 1691–1693 (2020). 32929268 10.1038/s41591-020-1083-1

[41] K. A. Callow, H. F. Parry, M. Sergeant, D. A. Tyrrell, The time course of the immune response to experimental coronavirus infection of man. Epidemiol. Infect. 105, 435–446 (1990). 10.1017/S0950268800048019 2170159 PMC2271881

[42] L. A. Jackson, E. J. Anderson, N. G. Rouphael, P. C. Roberts, M. Makhene, R. N. Coler, M. P. McCullough, J. D. Chappell, M. R. Denison, L. J. Stevens, A. J. Pruijssers, A. McDermott, B. Flach, N. A. Doria-Rose, K. S. Corbett, K. M. Morabito, S. O’Dell, S. D. Schmidt, P. A. Swanson 2nd, M. Padilla, J. R. Mascola, K. M. Neuzil, H. Bennett, W. Sun, E. Peters, M. Makowski, J. Albert, K. Cross, W. Buchanan, R. Pikaart-Tautges, J. E. Ledgerwood, B. S. Graham, J. H. Beigel; mRNA-1273 Study Group, An mRNA Vaccine against SARS-CoV-2 - Preliminary Report. N. Engl. J. Med. 383, 1920–1931 (2020). 32663912 10.1056/NEJMoa2022483 PMC7377258

[43] M. J. Mulligan, K. E. Lyke, N. Kitchin, J. Absalon, A. Gurtman, S. Lockhart, K. Neuzil, V. Raabe, R. Bailey, K. A. Swanson, P. Li, K. Koury, W. Kalina, D. Cooper, C. Fontes-Garfias, P.-Y. Shi, Ö. Türeci, K. R. Tompkins, E. E. Walsh, R. Frenck, A. R. Falsey, P. R. Dormitzer, W. C. Gruber, U. Şahin, K. U. Jansen, Phase I/II study of COVID-19 RNA vaccine BNT162b1 in adults. Nature 586, 589–593 (2020). 10.1038/s41586-020-2639-4 32785213

[44] P. M. Folegatti, K. J. Ewer, P. K. Aley, B. Angus, S. Becker, S. Belij-Rammerstorfer, D. Bellamy, S. Bibi, M. Bittaye, E. A. Clutterbuck, C. Dold, S. N. Faust, A. Finn, A. L. Flaxman, B. Hallis, P. Heath, D. Jenkin, R. Lazarus, R. Makinson, A. M. Minassian, K. M. Pollock, M. Ramasamy, H. Robinson, M. Snape, R. Tarrant, M. Voysey, C. Green, A. D. Douglas, A. V. S. Hill, T. Lambe, S. C. Gilbert, A. J. Pollard; Oxford COVID Vaccine Trial Group, Safety and immunogenicity of the ChAdOx1 nCoV-19 vaccine against SARS-CoV-2: A preliminary report of a phase 1/2, single-blind, randomised controlled trial. Lancet 396, 467–478 (2020). 10.1016/S0140-6736(20)31604-4 32702298 PMC7445431

[45] C. Keech, G. Albert, I. Cho, A. Robertson, P. Reed, S. Neal, J. S. Plested, M. Zhu, S. Cloney-Clark, H. Zhou, G. Smith, N. Patel, M. B. Frieman, R. E. Haupt, J. Logue, M. McGrath, S. Weston, P. A. Piedra, C. Desai, K. Callahan, M. Lewis, P. Price-Abbott, N. Formica, V. Shinde, L. Fries, J. D. Lickliter, P. Griffin, B. Wilkinson, G. M. Glenn, Phase 1-2 Trial of a SARS-CoV-2 Recombinant Spike Protein Nanoparticle Vaccine. N. Engl. J. Med. (2020). 32877576 10.1056/NEJMoa2026920 PMC7494251

[46] C. A. Hogan, M. K. Sahoo, B. A. Pinsky, Sample Pooling as a Strategy to Detect Community Transmission of SARS-CoV-2. JAMA 323, 1967–1969 (2020). 10.1001/jama.2020.5445 32250394 PMC7136853

[47] V. M. Corman, O. Landt, M. Kaiser, R. Molenkamp, A. Meijer, D. K. Chu, T. Bleicker, S. Brünink, J. Schneider, M. L. Schmidt, D. G. Mulders, B. L. Haagmans, B. van der Veer, S. van den Brink, L. Wijsman, G. Goderski, J.-L. Romette, J. Ellis, M. Zambon, M. Peiris, H. Goossens, C. Reusken, M. P. Koopmans, C. Drosten, Detection of 2019 novel coronavirus (2019-nCoV) by real-time RT-PCR. Euro Surveill. 25, (2020). 10.2807/1560-7917.ES.2020.25.3.2000045 31992387 PMC6988269

[48] D. Stadlbauer, F. Amanat, V. Chromikova, K. Jiang, S. Strohmeier, G. A. Arunkumar, J. Tan, D. Bhavsar, C. Capuano, E. Kirkpatrick, P. Meade, R. N. Brito, C. Teo, M. McMahon, V. Simon, F. Krammer, SARS-CoV-2 Seroconversion in Humans: A Detailed Protocol for a Serological Assay, Antigen Production, and Test Setup. Curr. Protoc. Microbiol. 57, e100 (2020). 10.1002/cpmc.100 32302069 PMC7235504

[49] A. E. Powell, K. Zhang, M. Sanyal, S. Tang, P. A. Weidenbacher, S. Li, T. D. Pham, J. E. Pak, W. Chiu, P. S. Kim, A single immunization with spike-functionalized ferritin vaccines elicits neutralizing antibody responses against SARS-CoV-2 in mice. bioRxiv 2020.08.28.272518 (2020). 10.1101/2020.08.28.272518 33527087 PMC7805605

[50] R Core Team, (2013). R: A language and environment for statistical computing. R Foundation for Statistical Computing, Vienna, Austria. URL: http://www.R-project.org/.

